# *Staphylococcus aureus* Bacteraemia in a Tropical Setting: Patient Outcome and Impact of Antibiotic Resistance

**DOI:** 10.1371/journal.pone.0004308

**Published:** 2009-01-30

**Authors:** Emma K. Nickerson, Maliwan Hongsuwan, Direk Limmathurotsakul, Vanaporn Wuthiekanun, Krupal R. Shah, Pramot Srisomang, Weera Mahavanakul, Therapon Wacharaprechasgul, Vance G. Fowler, T. Eoin West, Nitaya Teerawatanasuk, Harald Becher, Nicholas J. White, Wirongrong Chierakul, Nicholas P. Day, Sharon J. Peacock

**Affiliations:** 1 Mahidol-Oxford Tropical Medicine Research Unit, Mahidol University, Bangkok, Thailand; 2 Centre for Clinical Vaccinology and Tropical Medicine, Nuffield Department of Clinical Medicine, University of Oxford, Oxford, United Kingdom; 3 Duke University Medical Center, Durham, North Carolina, United States of America; 4 Department of Pediatrics, Sappasithiprasong Hospital, Ubon Ratchathani, Thailand; 5 Department of Medicine, Sappasithiprasong Hospital, Ubon Ratchathani, Thailand; 6 Department of Radiology, Sappasithiprasong Hospital, Ubon Ratchathani, Thailand; 7 Department of Medicine, Harborview Medical Center, University of Washington, Seattle, Washington, United States of America; 8 Department of Microbiology, Sappasithiprasong Hospital, Ubon Ratchathani, Thailand; 9 Department of Cardiology, John Radcliffe Hospital, Oxford, United Kingdom; Columbia University, United States of America

## Abstract

**Background:**

Most information on invasive *Staphylococcus aureus* infections comes from temperate countries. There are considerable knowledge gaps in epidemiology, treatment, drug resistance and outcome of invasive *S. aureus* infection in the tropics.

**Methods:**

A prospective, observational study of *S. aureus* bacteraemia was conducted in a 1000-bed regional hospital in northeast Thailand over 1 year. Detailed clinical data were collected and final outcomes determined at 12 weeks, and correlated with antimicrobial susceptibility profiles of infecting isolates.

**Principal Findings:**

Ninety-eight patients with *S. aureus* bacteraemia were recruited. The range of clinical manifestations was similar to that reported from temperate countries. The prevalence of endocarditis was 14%. The disease burden was highest at both extremes of age, whilst mortality increased with age. The all-cause mortality rate was 52%, with a mortality attributable to *S. aureus* of 44%. Methicillin-resistant *S. aureus* (MRSA) was responsible for 28% of infections, all of which were healthcare-associated. Mortality rates for MRSA and methicillin-susceptible *S. aureus* (MSSA) were 67% (18/27) and 46% (33/71), respectively (p = 0.11). MRSA isolates were multidrug resistant. Only vancomycin or fusidic acid would be suitable as empirical treatment options for suspected MRSA infection.

**Conclusions:**

*S. aureus* is a significant pathogen in northeast Thailand, with comparable clinical manifestations and a similar endocarditis prevalence but higher mortality than industrialised countries. *S. aureus* bacteraemia is frequently associated with exposure to healthcare settings with MRSA causing a considerable burden of disease. Further studies are required to define setting-specific strategies to reduce mortality from *S. aureus* bacteraemia, prevent MRSA transmission, and to define the burden of *S. aureus* disease and emergence of drug resistance throughout the developing world.

## Introduction

The published literature on invasive *Staphylococcus aureus* disease is heavily skewed towards industrialised temperate countries, where it represents a major cause of community- and hospital-acquired infection [1], [2]. Clinical management has been complicated by the increasing proportion of infections caused by methicillin-resistant *S. aureus* (MRSA), a problem that was initially limited to hospital-adapted strains but that has now extended to community-associated strains [3], [4]. Attributable mortality rates for *S. aureus* bacteraemia (SAB) in the developed world are typically up to 30% [5]–[7], and the costs of treating nosocomial infection, screening for MRSA carriage and changes in prescribing practices to include cover for MRSA are considerable. This situation has attracted huge public and media attention and receives significant research funding.

This situation strikes a sharp contrast with that in the developing world, where *S. aureus* is considered to be a less important cause of morbidity and mortality than other infectious diseases such as pneumococcal infections, malaria, HIV infection and tuberculosis. There are few studies of *S. aureus* infection in tropical countries that define disease epidemiology, the nature of clinical manifestations, outcome and drug resistance patterns. Yet the spread of pandemic clones of MRSA has major implications for the developing world, where facilities to determine antimicrobial resistance are lacking, expensive antibiotics required to treat MRSA are often not available and hospital infection control policies are limited. The availability of over-the-counter antibiotics in much of the tropical world is likely to be a major driver of drug resistance.

Here, we begin to fill our knowledge gaps with a study that describes the epidemiology, treatment and outcome from *S. aureus* bacteraemia in northeast Thailand, together with the incidence, place of acquisition and impact of MRSA infections.

## Methods

### Patients and Clinical methods

The Ethical and Scientific Review sub-committee of the Royal Thai Government Ministry of Public Health, and the Oxford Tropical Research Ethics Committee both approved the study. A prospective, observational study was conducted at Sappasithiprasong Hospital in northeast Thailand for a period of 1 year, from November 2006 to November 2007. This 1000-bed regional hospital has a catchment of 2 million people, the majority of whom are rice farmers and their families. The hospital provides a comprehensive clinical and laboratory service. Potential study patients were identified by daily screening of the blood culture results in the hospital diagnostic microbiology laboratory. Patients of any age with at least one clinically significant blood culture positive for a pure growth of *S. aureus* were enrolled into the study after providing written informed consent. Following recruitment, study investigators visited inpatients daily until discharge to record progress and management. Data were recorded on standardised forms adapted from those developed by Fowler et al [8]. Clinical care was provided by the hospital physicians, independent of the research team. Study-specific investigations were a repeat blood culture taken 48–96 hours after the first culture positive for *S. aureus* and a transthoracic echocardiogram. Results of study investigations were given to hospital physicians caring for the patient. Echocardiograms on patients aged less than 14 years were performed by a paediatric echocardiographer (T.W.), while echocardiograms on the remainder were performed and/or reviewed by study investigators trained in adult echocardiography (E.K.N., W.M., H.B.). Final outcome was determined 12 weeks from the date the first culture positive for *S. aureus* was taken using a standardised telephone questionnaire.

### Definitions

Community-acquired infection was defined as admission to hospital with an illness consistent with invasive *S. aureus* disease and a positive *S. aureus* blood culture. Nosocomial infection was defined as a positive *S. aureus* blood culture taken more than 48 hours after admission for another condition. Non-nosocomial healthcare-associated infection was defined as community-acquired infection above but in an individual who had been in contact with healthcare services in the preceding year, using the criteria described by Fowler et al [8]. Sites of *S. aureus* infection were established based on examination findings and referral to the medical notes, including investigation reports and operation notes. A history of an underlying chronic medical condition was recorded if documented in the medical notes. Outcomes at 12 weeks were defined as: (i) cure - clinically improved and no additional sites of infection present or suspected; (ii) unresolved infection - persistent features of infection with or without persistent positive cultures; (iii) death attributable to *S. aureus* - when death was due to *S. aureus* infection in a previously healthy individual or when *S. aureus* hastened death in the presence of an underlying condition such as cancer; or (iv) death due to other causes - when the *S. aureus* infection did not appear to contribute to death.

### Laboratory methods

Antibiotic susceptibilities were performed by the hospital microbiology laboratory using the disk diffusion method [9]. Susceptibilities reported to hospital physicians were penicillin, oxacillin, cefazolin, erythromycin, clindamycin and trimethoprim-sulphamethoxazole for methicillin-susceptible *S. aureus* (MSSA). For MRSA isolates, susceptibilities to vancomycin and fusidic acid were added. A total of 81/98 *S. aureus* isolates were available for further study, re-identified and stored at −80°C. An extended antibiotic susceptibility profile was determined in our research laboratory at the hospital using the disk diffusion method [9] to establish susceptibilities to other antibiotic agents with potential activity against the clinical isolates. This panel included cefoxitin, chloramphenicol, ciprofloxacin, clindamycin, erythromycin, fusidic acid, gentamicin, mupirocin, netilmicin, penicillin, rifampicin, trimethoprim-sulphamethoxazole, tetracycline and vancomycin. Isolates that were resistant to cefoxitin by disk diffusion were evaluated by oxacillin and vancomycin E-tests (AB Biodisk). Isolates were designated as MRSA based on an oxacillin E-test for 81 isolates, and based on the oxacillin disk diffusion assay performed by the hospital laboratory for the 17 isolates that were not available for further testing.

### Statistical analysis

Data were double entered into a database. Data analysis was performed using Stata software version 9 (StataCorp, College Station, Texas). Categorical data were analysed using Fisher's exact test and for continuous data the Mann-Whitney U test was used. Reported p values are two-tailed. Kaplan-Meier survival curves were plotted for attributable and non-attributable deaths, and for *S. aureus* attributable deaths in children and adults (non-attributable deaths censored). These survival curves were compared using the log rank test.

## Results

### Patient characteristics

A total of 106 patients fulfilled the inclusion criteria during the study period. Of these, 98 patients were recruited and 8 patients could not be studied either because they declined to consent to participate in the study (n = 6) or because they returned home to Lao PDR (across the Mekong River from Thailand) before the culture became positive and follow up was not possible (n = 2). The age of enrolled patients ranged from 1 day to 92 years (median 39 years). There were 61 (62%) adult and 37 paediatric patients (less than 18 years) (Table 1). A history of one or more underlying chronic medical conditions was documented in 57 cases (58%), with cardiac disease accounting for the greatest proportion (20 cases). A site of infection was identified in 59 (60%) patients.

**Table 1:** Patient characteristics.

	All patients (n = 98)
	*Number of patients (percent)*
**Demographics**	
Age (years), median (interquartile range)	39 (9–65)
Sex (male)	57 (58%)
Thai nationality	96 (98%)*1
**Co-morbidities**	
Prematurity/ Very low birth weight	5 (5%)
Intravenous drug use	4 (4%)
Underlying medical conditions*2	57 (58%)
- Cardiac disease	20 (20%)
- Diabetes mellitus	12 (12%)
- Renal disease	11 (11%)
- Immunosuppression*3	11 (11%)
- Lung disease	6 (6%)
**Place of acquisition**	
Community-acquired	44 (45%)
Nosocomial	40 (41%)
Non-nosocomial healthcare-associated	14 (14%)
**Types of disease**	
No identified site of infection	39 (40%)
1 identified site of infection	46 (47%)
>1 identified site of infection	13 (13%)

Sites of infection*4	All sites (n = 73)
	*Number of sites (percent)*
Skin and soft tissue infections	18 (25%)
Pneumonia	12 (16%)
Device-related infections*5	10 (14%)
Endocarditis*6	8 (11%)
Septic arthritis	7 (10%)
Post-operative mediastinitis*7	4 (5%)
Other lung infections*8	4 (5%)
Solid organ abscess*9	3 (4%)
Osteomyelitis	2 (3%)
Pyomyositis	2 (3%)
Diabetic foot infections	2 (3%)
Urinary tract infection	1 (1%)

*1 Two patients were from Lao PDR.

*2 History of any underlying chronic medical condition documented in the medical notes.

*3 Immunosuppression from chemotherapy (n = 4), untreated leukaemia (n = 1), HIV (n = 3, none of whom were on anti-retroviral therapy or any prophylactic antibiotics) or immunosuppressive medication including prednisolone >30 mg/day for >1 week (n = 4; one of these additionally on chemotherapy).

*4 Denominator is number of sites (n = 73) since some patients had >1 site of infection.

*5 Intravenous catheters (central n = 3, peripheral n = 2, umbilical n = 1), pacemakers (n = 3) and arteriovenous graft (n = 1).

*6 Vegetations on transthoracic echocardiography (n = 7); or strong clinical evidence in intravenous drug user (n = 1).

*7 Following mitral valve replacement (n = 3), or coronary artery bypass graft (n = 1).

*8 Empyema (n = 2), septic emboli to the lungs (n = 1), lung abscesses (n = 1).

*9 Liver (n = 2), spleen (n = 1).

Repeat blood cultures were performed within 48–96 hours of the first culture positive for *S. aureus* in 57 patients (58%, representing 79% of patients surviving to 4 days), of which 11 (19%) were still positive. After this time period, 56 further blood cultures were taken from 23 patients, which identified an additional 3 patients with persistently positive blood cultures. No vegetations were seen on transthoracic echocardiography performed on 11 of these 14 patients with persistently positive cultures. MRSA was responsible for persistently positive cultures significantly more often than MSSA infection (30% versus 8% respectively (p = 0.02)). Eight of 14 patients (57%) were receiving antibiotic therapy to which the organism was susceptible *in vitro* at the time the repeat blood culture was taken.

### Prevalence of endocarditis

Echocardiography was performed in 49 out of 98 patients (50%). Although the aim of the study was to perform echocardiography on all patients, the remaining 49 patients were either discharged or died before an echocardiogram could be performed, were too sick to be taken to the echocardiography room, had a contraindication to transthoracic echocardiography, or the echocardiography machine or trained personnel were not available. However, there were no significant differences between those who did and did not undergo echocardiography in terms of pre-disposing cardiac conditions, co-morbidities, immunocompromise, age or presence of prosthetic material (data not shown). Seven of the 49 patients (14%) undergoing echocardiography had vegetations visualized. The affected valves were mitral (n = 3), aortic (n = 3) and tricuspid (n = 1). Two patients had definite endocarditis and 5 had possible endocarditis by modified Duke criteria [10], since taking 2 blood culture bottles is uncommon in the hospital. Among these 7 patients with endocarditis, 1 died. One additional patient with a history of injection drug use met clinical criteria for endocarditis, but died before echocardiography could be performed.

### Healthcare exposure

Over half of the infections (54%) were either nosocomial or non-nosocomial healthcare-associated. Nosocomial infections were most common in those aged less than 1 year old, accounting for 94% of cases. Infections related to devices were the third most common site of infection, accounting for 14% of all sites.

### Antibiotic resistance and therapy

MRSA accounted for 28% (n = 27) of SAB, with no significant difference in rate between adults and children (26% versus 30% respectively; p = 0.82). The MRSA cases were either nosocomial (n = 21, 78%) or non-nosocomial healthcare-associated (n = 6, 22%). Of the 71 MSSA cases, 44 (62%) were community-acquired, 8 (11%) were non-nosocomial healthcare-associated, and 19 (27%) were nosocomial. There was a significant difference in MRSA rates on the intensive care units compared with the general wards: 57% versus 16%, respectively (p<0.001). The impact of MRSA on effective prescribing of antibiotic therapy is summarised in Table 2. Patients infected with MSSA were more likely than patients infected with MRSA to receive an antibiotic to which the organism was susceptible before the culture results became available (67/71 (94%) versus 4/27 (15%) respectively, p<0.001). The median number of days to starting antibiotic therapy to which the organism was susceptible *in vitro* was 0 (interquartile range (IQR) 0–0) days and 3 (IQR, 2–4) days for MSSA and MRSA, respectively (p<0.001). Once culture results were available, 70 out of 73 survivors (96%) received an antibiotic that covered the infecting isolate. The median duration of parenteral treatment given to survivors from the day the first culture positive for *S. aureus* was taken was 17 (IQR 12–24) days, with no difference in duration for patients infected with MSSA versus MRSA (p = 0.26). All MRSA isolates were susceptible to vancomycin by E-test (minimum inhibitory concentration ≤2 µg/ml). Of the 17 patients with normal renal function who received vancomycin, 13 (76%) received adequate vancomycin doses by manufacturer's recommendations. Measurement of vancomycin drug levels was not available in the hospital.

**Table 2:** Impact of MRSA on effective antibiotic prescribing.

	All patients (n = 98)	MSSA*1 (n = 71)	MRSA*1 (n = 27)	p value*2
	*Number of patients (percent)*			
**Pre-culture results**				
Antibiotic therapy prescribed	93 (95%)	67 (94%)	26 (96%)	>0.99
Infecting strain of *S. aureus* susceptible to prescribed antibiotic	71 (72%)	67 (94%)	4 (15%)	**<0.001**
Optimal antibiotic therapy*3	25 (26%)	23 (32%)	2 (7%)	**0.01**

Post-culture results*4	All patients (n = 73)	MSSA (n = 50)	MRSA (n = 23)	
Antibiotic therapy prescribed	72 (99%)	49 (98%)	23 (100%)	>0.99
Infecting strain of *S. aureus* susceptible to prescribed antibiotic	70 (96%)	48 (96%)	22 (96%)	>0.99
Optimal antibiotic therapy*3	53 (73%)	32 (64%)	21 (91%)	**0.02**

*1 MSSA, methicillin-susceptible *S. aureus*; MRSA, methicillin-resistant *S. aureus*.

*2 p value comparing MSSA and MRSA groups.

*3 Optimal therapy defined as cloxacillin for MSSA infection and vancomycin for MRSA infection. Alternative therapy used included ceftriaxone, cefazolin, cefoxitin, ceftazidime, augmentin and ampicillin combined with gentamicin.

*4 Denominator is patients who survived to day of culture result. A total of 25 patients (21 with MSSA and 4 with MRSA) died or were discharged moribund prior to culture results becoming available.

Antibiotic resistance patterns are summarised in Table 3. Over 90% of MRSA isolates were resistant to ciprofloxacin, erythromycin, gentamicin, netilmicin, tetracycline and trimethoprim-sulphamethoxazole, and 65% were resistant to clindamycin. Vancomycin and fusidic acid had the lowest rates of resistance.

**Table 3:** Antibiotic resistance rates for infecting isolates.

Antibiotic	All isolates (n = 81)	MSSA*1 (n = 58)	MRSA*1 (n = 23)
	Number of resistant isolates (%)		
Chloramphenicol	8 (10%)	4 (7%)	4 (17%)
Ciprofloxacin	22 (27%)	1 (2%)	21 (91%)
Clindamycin	16 (20%)	1 (2%)	15 (65%)
Erythromycin	25 (31%)	2 (3%)	23 (100%)
Fusidic acid	42 (52%)	39 (67%)	3 (13%)
Gentamicin	23 (28%)	0	23 (100%)
Mupirocin	7 (9%)	2 (3%)	5 (22%)
Netilmicin	21 (26%)	0	21 (91%)
Penicillin	80 (99%)	57 (98%)	23 (100%)
Rifampicin	8 (10%)	0	8 (35%)
Tetracycline	56 (69%)	34 (59%)	22 (96%)
Trimethoprim-sulphamethoxazole	22 (27%)	0	22 (96%)
Vancomycin	0	0	0

*1 MSSA, methicillin-susceptible *S. aureus*; MRSA, methicillin-resistant *S. aureus*.

### Mortality

**Figure 1:**
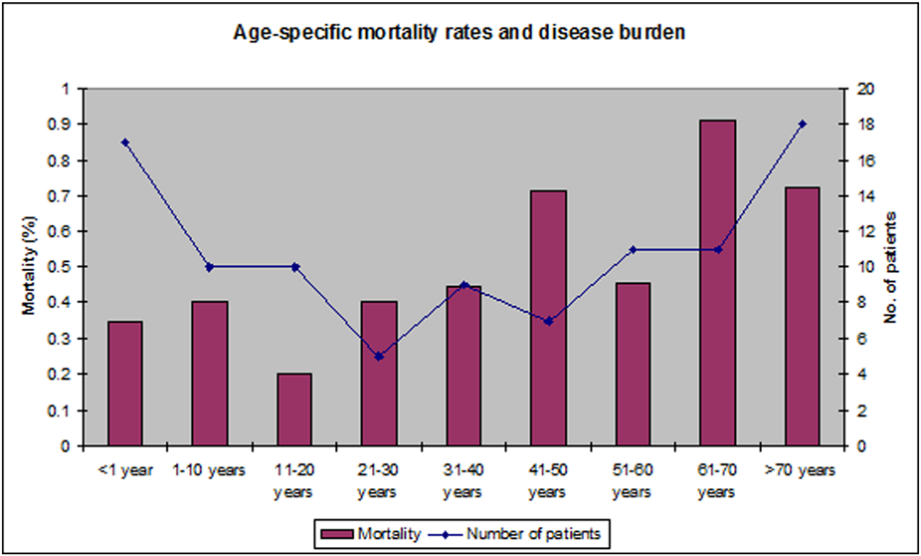
The burden of S. aureus bacteraemia is highest at the extremes of age, whilst mortality increased with age. Age distribution and age-specific mortality rates and disease burden for 98 cases of *S. aureus* bacteraemia studied in Ubon Ratchathani, NE Thailand. Overall mortality increased significantly with age (p<0.001), analysing age as a continuous variable.

**Figure 2:**
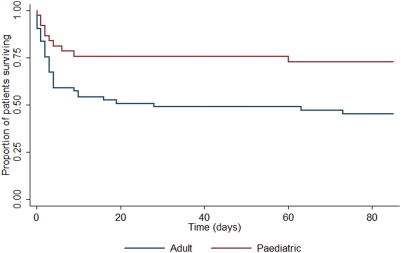
Survival from *S. aureus* bacteraemia is worse in adults than in children. Kaplan-Meier survival curves comparing adult and paediatric patients with respect to *S. aureus* attributable deaths (p = 0.01). Non-attributable deaths were censored.

**Figure 3:**
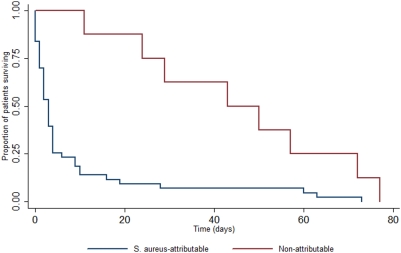
*S. aureus* attributable deaths occur more rapidly than non-attributable deaths. Kaplan-Meier survival curves comparing *S. aureus* attributable deaths and non-attributable deaths (p = 0.001).

The overall mortality rate at 12 weeks was 52% (n = 51), and the *S. aureus* attributable mortality rate was 44% (n = 43). There was insufficient information to determine the cause of death in 1 case. Most deaths (39/51, 76%) occurred in hospital or at home on the day of discharge, as relatives commonly take moribund patients home to die (n = 12). *S. aureus* bacteraemia accounted for 1% of all in-patient deaths at Sappasithiprasong hospital during the study period. Overall mortality increased significantly with age (p<0.001) (Figure 1). There was a significant difference between the overall mortality rate in children compared to adults (32% versus 64%, p = 0.003). The association between overall mortality and increasing age remained highly significant when adjusted for underlying co-morbidities or immunosuppression (p = 0.001). The mortality rates for MRSA and MSSA were 67% (18/27) and 46% (33/71), respectively (p = 0.11). There was no significant difference in mortality between patients with and without identified sites of infection, (47% and 59% respectively, p = 0.31). Mortality amongst patients with repeated blood cultures positive for *S. aureus* was 43% (6/14). The median number of days to death in those patients whose death was attributable to *S. aureus* was 3 (IQR, 1–6) days compared with 47 (IQR, 25–68) days in those with non-attributable deaths (p<0.001). Significant differences were noted on survival curve analysis of attributable mortality rates in adults and children (p = 0.01) (Figure 2), and in the timing of death for *S. aureus* attributable and non-attributable deaths (p = 0.001) (Figure 3). There was no difference in times to death comparing patients infected with MSSA versus MRSA (data not shown). At 12 weeks, 44 patients (45%) were cured, and 3 patients (3%) had unresolved infection (empyema thoracis, infected pacemaker, and osteomyelitis).

## Discussion

This study shows that SAB is an important cause of morbidity and mortality in northeast Thailand. The all-cause and *S. aureus*-attributable mortality rates in our study, 52% and 44% respectively, are considerably higher than mortality rates for SAB reported from temperate industrialised countries [5]–[7]. A study of SAB conducted in the USA by Fowler et al [8] found an all-cause and attributable mortality of 22% (157/724) and 12% (86/724), respectively, but this excluded deaths that occurred before culture results were available. The addition of these cases would have given an all-cause and *S. aureus*-attributable mortality rate of 33% and 23%, respectively. Published studies of all-cause bacteraemia in Asia have identified *S. aureus* as a major cause of bacteraemia, accounting for both community-acquired and hospital-acquired disease [11]–[13]. If our data showing that *S. aureus* accounts for 1% of all in-patient deaths is representative of this populous region (half the world's population lives within 2000 miles of northeast Thailand), then *S. aureus* would be a major contributor to preventable mortality worldwide. To our knowledge this is the first published prospective study to focus on SAB in tropical Asia. This is noteworthy because papers describing all-cause bacteraemia in Asia rarely give details by causative organism, resulting in an under-appreciation of the morbidity and mortality burden due to *S. aureus* in the tropics. Sappasithiprasong Hospital is the regional hospital for the province with referrals from clinics and hospitals in the province, but also provides primary care services to a large local population so accurate estimates of disease incidence were not possible.

In our study, SAB was most common at the extremes of age, a similar pattern to that described in a temperate industrialised country [14], [15]. Among the 17 patients under 1 year of age, 16 (94%) had hospital-acquired infections and nearly a third (29%) were premature or very low birth weight babies. This suggests the particular vulnerability to acquiring nosocomial SAB of children less than 1 year of age requiring multiple interventions and prolonged stays in hospital. A higher incidence in those aged under 1 year and the predominance of nosocomial infections in this age group has been described in Denmark [14]. The increase in numbers of cases and mortality with age also mirror the rise in co-morbidities with age, as noted in Denmark [15].

The broad range of clinical manifestations seen in patients with SAB in industrialised countries was observed in this series. Our findings suggest that the burden of *S. aureus* disease in the tropics exceed current perceptions, and demonstrate that serious invasive infections are common. This has an important bearing on antimicrobial therapy because management of deep infections and bacteraemia requires effective antimicrobial therapy whereas drainage of superficial pus collections can result in cure irrespective of antibiotic therapy [16].

The rapid deterioration and short median time to death (3 days) of patients dying from *S. aureus* septicaemia meant that echocardiograms could not be performed in half of the patients. However, the prevalence of echocardiographically-confirmed endocarditis of 14% (7/49) is comparable to that seen in industrialised countries [8], [17], which has important implications for many tropical countries where restricted or delayed access to echocardiography is liable to be an issue. Our prevalence may be an underestimate since those patients who died earlier may have been more likely to have endocarditis. A quarter of the patients with endocarditis died, which is at the lower end of the mortality range reported for *S. aureus* endocarditis (25–47%) [18] and may be a further indication that a number of patients who died prior to echocardiography had undiagnosed endocarditis. The heart valves affected by endocarditis were predominantly left-sided, which is in keeping with the low number of intravenous drug users in our study. Half the patients with endocarditis were teenagers, which is a younger age group than typically seen in industrialised temperate countries [18], [19] but is described in other tropical countries [20], [21], often as a result of rheumatic heart disease and uncorrected congenital heart disease. However, none of our patients with endocarditis had known valvular abnormalities.

MRSA was responsible for nearly one third of cases of SAB, all of which were healthcare-associated. Although community-associated MRSA is a major problem elsewhere, we find no evidence for this in our setting where a clone defined by multilocus sequence typing as sequence type 239 predominates [22]. Over half of our patients had healthcare-associated infections which suggests that SAB in Thailand is strongly related to healthcare, mirroring industrialised countries [23], [24]. Although there are comprehensive hospital infection control guidelines in Sappasithiprasong hospital, implementing these is difficult due to a bed occupancy rate that often exceeds 100% and a lack of infrastructure, such as a scarcity of isolation rooms and only two hand wash basins on each of the general wards. However, alcohol hand rub is available in the intensive care units. Addressing such infection control measures would require a significant increase in investment, although this would be offset by reducing the expense of nosocomial infections. Our finding that over half the infections were healthcare-associated indicates that hospital infection control is an important area for clinical, microbiological and economic research if improvements are to be made.

Significant delays in receiving effective antibiotic therapy were seen with MRSA bacteraemia compared with MSSA bacteraemia. Vancomycin is available in this setting but is not used in the empiric regimen for a patient with suspected bacterial sepsis unless they are already known to be MRSA positive. Monitoring of vancomycin levels is not possible at Sappasithiprasong Hospital, which may lead doctors to give lower doses to reduce the perceived risk of toxicity. The mortality rates for patients with MRSA who received effective and ineffective empirical antibiotics were 0% and 69%, respectively, indicating the importance of early effective treatment. There was a median delay in effective antibiotic therapy of 3 days for MRSA patients. Delays in treating SAB are known to have an adverse effect on outcome [25]. The common usage of ampicillin and gentamicin as empirical therapy in children under the age of 1 year should be revised in light of our finding that 53% of SAB cases in this age group were MRSA and all MRSA strains were resistant to gentamicin. Although alternative antibiotics to vancomycin may be appropriate therapy for MRSA, in our setting the high rates of resistance found on susceptibility testing indicate that these alternatives would not be effective.

This study has demonstrated that *S. aureus* is a significant pathogen in northeast Thailand, with comparable clinical manifestations and a similar endocarditis prevalence but higher mortality than industrialised countries. The factors contributing to this high death rate, such as delayed presentation to hospital and the early management of sepsis, require further evaluation. The majority of infections were associated with exposure to healthcare settings and MRSA was associated with a considerable burden of disease and a high mortality. Revisions to the empirical prescribing practices to include MRSA therapy could be associated with significant benefit. An initiative to raise the profile of infection control is needed, together with work to characterise the burden and causes of hospital-acquired infections, including patient-to-patient transmission of MRSA, such that cost-effective solutions can be devised appropriate to this setting.
